# Silk/Rayon Webs and Nonwoven Fabrics: Fabrication, Structural Characteristics, and Properties

**DOI:** 10.3390/ijms23147511

**Published:** 2022-07-06

**Authors:** Yu Jeong Bae, Mi Jin Jang, In Chul Um

**Affiliations:** 1 Department of Biofibers and Biomaterials Science, Kyungpook National University, Daegu 41566, Korea; qodbwjd09@knu.ac.kr; 2 Preclinical Research Center, Daegu-Gyeongbuk Medical Innovation Foundation, Daegu 41061, Korea; mijin22@kmedihub.re.kr

**Keywords:** silk, rayon, nonwoven fabric, mechanical properties, cytocompatibility

## Abstract

Silk is a naturally occurring material and has been widely used in biomedical and cosmetic applications owing to its unique properties, including blood compatibility, excellent cytocompatibility, and a low inflammatory response in the body. A natural silk nonwoven fabric with good mechanical properties was recently developed using the binding property of sericin. In this study, silk/rayon composite nonwoven fabrics were developed to increase productivity and decrease production costs, and the effect of the silk/rayon composition on the structure and properties of the fabric was examined. The crystalline structure of silk and rayon was maintained in the fabric. As the silk content increased, the porosity and moisture regain of the silk/rayon web and nonwoven fabric decreased. As the silk content increased, the maximum stress of the web and nonwoven fabric increased, and the elongation decreased. Furthermore, the silk/rayon web exhibited the highest values of maximum stress and elongation at ~200 °C. Regardless of the silk/rayon composition, all silk/rayon nonwoven fabrics showed good cytocompatibility. Thus, the silk/rayon fabric is a promising material for cosmetic and biomedical applications owing to its diverse properties and high cell viability.

## 1. Introduction

Silk is a naturally occurring substance composed of two biomacromolecules: fibroin and sericin. It has been utilized as the finest textile material and is considered a promising biomaterial since the discovery of its useful properties. Silk exhibits good biocompatibility [1,2], excellent cytocompatibility [3], a low inflammatory response in the body [4,5], and biodegradability [6,7]. Because of these unique properties, silk has been studied for its application as a wound dressing [8,9], nerve conduit [10], tympanic membrane [11], membrane for guided bone regeneration [12,13,14,15], artificial heart value [16], and artificial mask pack [17,18].

For these bio-related applications, silk should be fabricated into various forms, including film, porous web, and gel. Among them, electrospun porous webs have been utilized extensively for these applications. To produce an electrospun silk web, many processes are required (degumming, dissolution, dialysis, drying, dissolution, and electrospinning). These procedures increase production costs and diminish quality consistency. Additionally, silk experiences a disruption of its highly crystallized structure and molecular degradation [19,20,21,22], resulting in a deterioration of its mechanical properties. These problems restrict the commercialization of silk for cosmetic and medical applications.

Recently, a new natural silk nonwoven fabric (i.e., without regeneration) was successfully fabricated and could be mass-produced using an electric winder system [23,24,25,26]. The fabrication of natural silk nonwoven fabric is straightforward (reeling, wetting, and hot pressing), and the resultant nonwoven fabric has better mechanical properties than electrospun web [23].

Silk is more expensive than other natural or nature-based fibers (such as cotton, wool, flax, and rayon). Although the production cost of silk nonwoven fabric is not considered to be a problem in biomedical applications, the production cost of silk nonwoven fabrics should be decreased to increase its applications in fields such as cosmetics and filters.

Rayon is a regenerated cellulose fiber created by dissolving and wet-spinning wood and cotton cellulose. It is biocompatible, hydrophilic, and less expensive than silk [27]. It has been extensively used in cosmetics (especially mask packs) and biomedical applications [28,29].

In this study, we fabricated a silk/rayon composite web and nonwoven fabric using an electric winder system and examined the effect of the silk/rayon composition on the structure and properties of the web and nonwoven fabric.

## 2. Results and Discussion

### 2.1. Structural Characteristics of Silk/Rayon Web and Nonwoven Fabric

**Figure 1:**
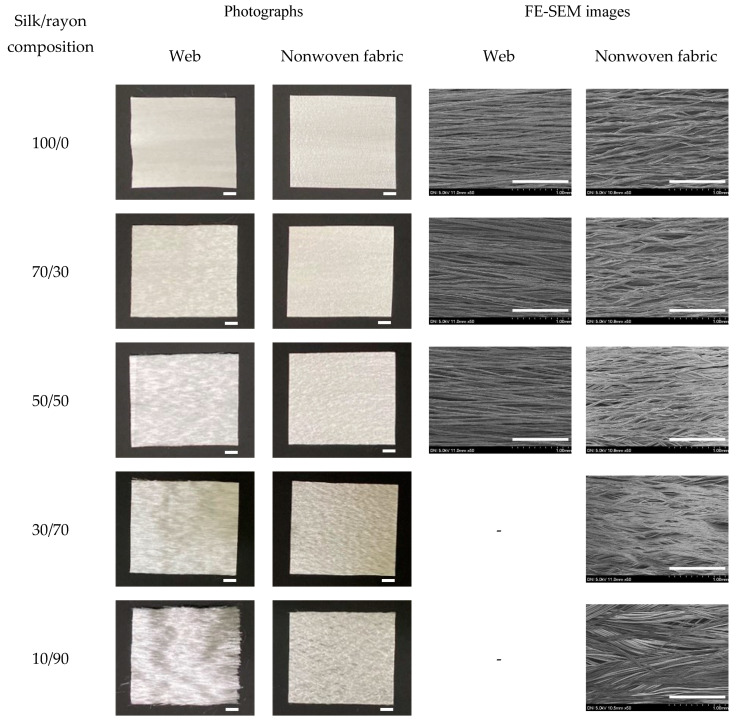
Photographs and FE-SEM images of the silk/rayon webs and nonwoven fabrics with different silk/rayon compositions. The white magnification bars in the photographs and the SEM images represent 1.0 cm and 1.0 mm, respectively.

Figure 1 shows the photographs and SEM images of the silk/rayon web and nonwoven fabric with various silk/rayon compositions. As shown in the figure, the web and nonwoven fabric with all tested compositions (100/0–10/90 silk/rayon) can be produced. As the rayon content increases, the surface of the web becomes more abrasive. Because of the heat and pressure applied during the hot press treatment of the web (i.e., the preparation of the nonwoven fabric), the surface roughness is considerably reduced, despite the silk and rayon filaments in the nonwoven fabric bending.

The roughness of the web and the bending of the filaments in the nonwoven fabric are due to the wetting and drying of the silk and rayon filament. Thus, the rayon filament becomes wet because it comes into contact with a wet silk filament reeled from a water bath in a roller. The wet silk and rayon filaments are dried after the reeling process. At this time, the silk/rayon web becomes coarse, and the water from the silk and rayon filaments is extracted. Silk and rayon filaments in the nonwoven fabric bend for the same reason. The silk and rayon filaments become wet because of the wet treatment. Subsequently, they are hot-pressed at a high temperature (200 °C). Therefore, the water in silk and rayon filaments evaporates rapidly, resulting in bent filaments in the nonwoven fabric. The rougher web and more curved filament in the nonwoven fabric with a higher rayon content may be due to rayon’s greater water-absorption capacity than silk [30]. That is, rayon retains more water during the wet treatment and loses more water, resulting in structures that are more rigid and bowed.

In the case of silk/rayon webs containing less than 50% silk, the binding of silk and rayon filaments was insufficient for SEM sampling. Owing to the improved binding between silk and rayon filaments, SEM was possible for all silk/rayon nonwoven compositions (100/0–10/90 silk/rayon). Thus, when silk was hot-pressed after a wet treatment, the binding effect of sericin increased [24,25,26,31].

The composite nonwoven fabric can be manufactured with a 90% rayon content, despite the absence of a binder in rayon. The silk/rayon composite nonwoven fabric can be produced with only a 10% silk content. As the silk filament contains 26% sericin, the silk/rayon nonwoven fabric can be fabricated with a sericin content of 2.6%. This indicates that various silk-based composite nonwoven fabrics can be prepared with a negligible amount of sericin (i.e., 2.6%). The cosmetic and biomedical industries require a range of capabilities. As numerous silk-based nonwovens with diverse compositional ratios can be manufactured, silk-based nonwovens are expected to be a promising material for biomedical and cosmetic applications.

**Figure 2:**
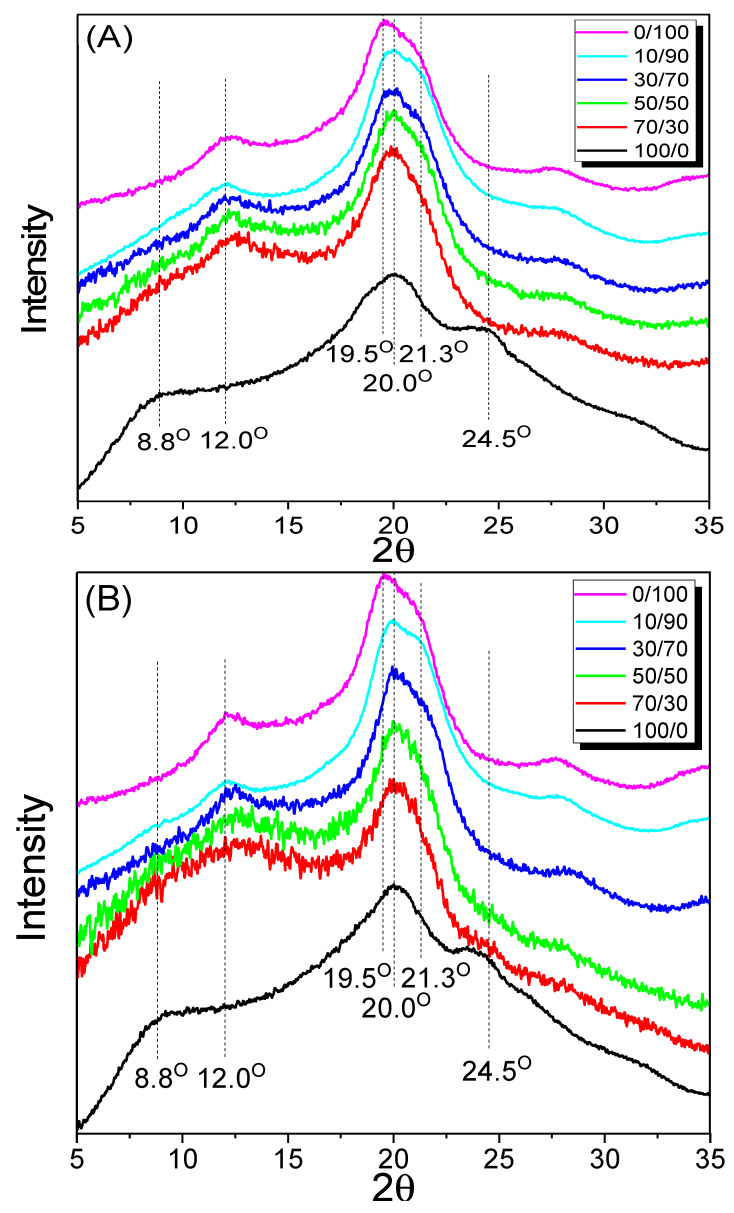
X-ray diffractograms of the (**A**) silk/rayon webs and (**B**) nonwoven fabrics with different silk/rayon (100/0–10/90) compositions. The 0/100 samples indicate rayon filaments in (**A**) and hot-pressed rayon filaments in (**B**) because rayon webs and nonwoven fabrics cannot be fabricated.

The crystalline structure of fibers and polymers affects their physical properties, and X-ray diffractometry studies have been conducted to investigate this [23,25,32]. Figure 2 shows the XRD results of the silk/rayon web and nonwoven fabric with varying silk contents. The 100% silk web exhibited β-sheet crystallite diffraction peaks at 2θ = 8.8°, 20.0°, and 24.5° [33,34]. The 100% rayon fibers exhibited peaks at 12.0°, 19.5°, and 21.3° owing to the cellulose II structure [35,36]. No discernible difference was observed between the web and nonwoven silk/rayon fabric. According to previous research, heat treatment induces a slight increase in the crystallization of silk and rayon [25,37]. Therefore, the additional crystallization of silk and rayon caused by the heat treatment was insignificant.

As the content of a component (i.e., silk or rayon) was increased, the characteristic peak of the component became more pronounced in both webs and nonwoven fabrics. This result can be easily rationalized as silk and rayon are physically separated in the web and nonwoven fabric. Therefore, one component cannot affect the crystallization of an opposing component.

**Figure 3:**
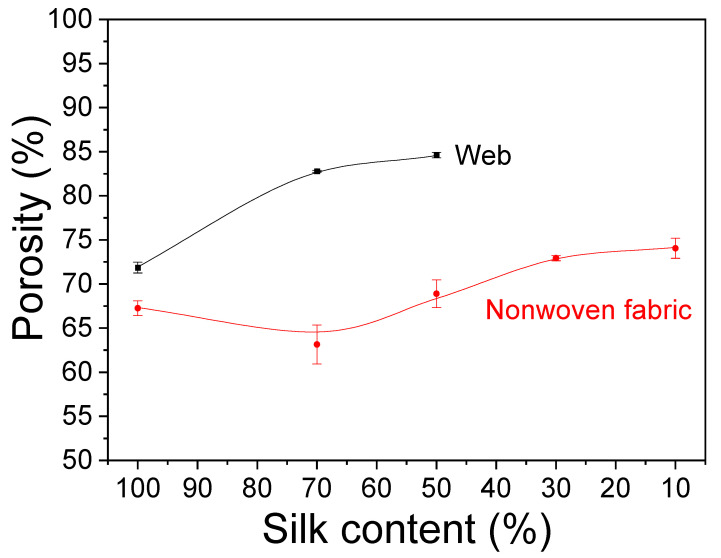
Porosity of the silk/rayon webs and the nonwoven fabrics with different silk contents.

The porous web and nonwoven fabric are widely used in cosmetic (e.g., mask pack) [17,18] and biomedical applications (e.g., wound dressing [8,9] and membrane for guided regeneration [12,13,14,15]) because they can hold more water [38] and allow cell adhesion and proliferation [39,40,41] through their pores. In addition, the porosity influences the mechanical properties of porous materials [32,42]. Therefore, porosity can be considered to be an important structural factor of the web and nonwoven fabric for biomedical and cosmetic applications. Figure 3 displays the results of measuring the porosity of the silk/rayon web and nonwoven fabric in this study.

The silk/rayon nonwoven fabrics had a lower porosity than the webs. As shown in Figure 1, this result is easily explicable as the hot-press treatment eliminates the space between the silk and rayon filaments, resulting in a more compact nonwoven morphology.

As the silk content decreased until it reached 50%, the porosity of the silk/rayon web increased. Owing to the insufficient adhesion between silk and rayon filaments, measuring the porosity in fabrics containing more than 50% rayon (i.e., the web became disassembled in measurement media) was impossible. The porosity of nonwoven fabric decreased slightly at a 70% silk content and then increased steadily with a decreasing silk content. There are two reasons for the increasing porosity of the web and nonwoven fabrics. First, in this study, the web and nonwoven fabric were produced by using silk filaments of three denier and rayon filaments of 72 denier. Owing to the increased denier (thickness) of rayon filament, more space was created between the filaments. With an increasing rayon content, the porosity of the web and nonwoven fabric increased (i.e., a decrease in the silk content). Another reason is the more robust structure of the web and nonwoven fabric with a higher rayon content. As shown in Figure 1, the filaments in the web and nonwoven fabric become more robust as the rayon content increases. An increased degree of ruggedness resulted in more space between filaments, thereby increasing porosity.

The porosity of the nonwoven fabric decreased slightly with a 70% silk content. Owing to the regular arrangement of fine silk filaments (three denier), the 100% silk web had a compact structure with low porosity. Before the hot-press treatment, the 100% silk web was wet-treated. During the wet treatment of the silk web, the water spread out the silk filaments, increasing the distance between them. This caused an increase in porosity. In contrast, silk/rayon (70/30) webs restricted the spread of silk filaments with water owing to the presence of rayon filaments. After the hot-press treatment, the varying degree of web spread was fixed. This caused the silk/rayon (70/30) nonwoven fabric to be less porous than the 100% silk nonwoven fabric.

### 2.2. Properties of Silk/Rayon Web and Nonwoven Fabric

**Figure 4:**
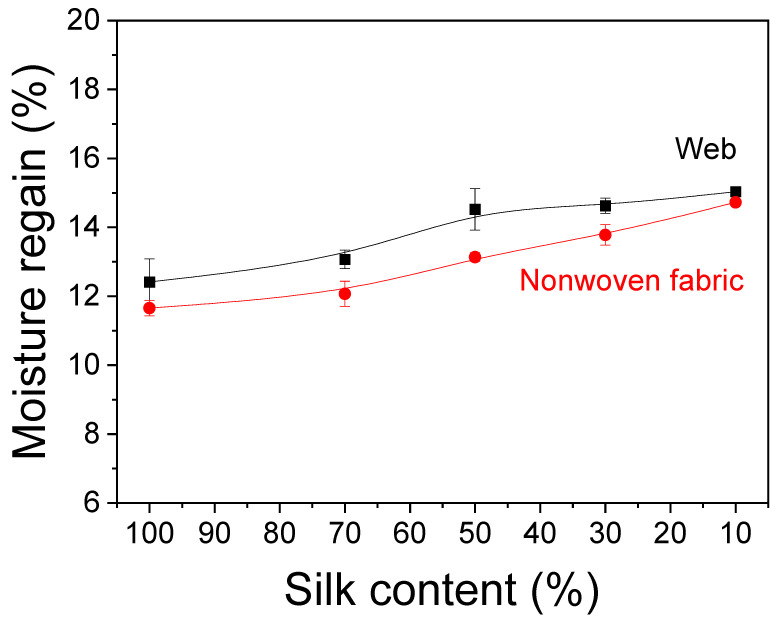
Moisture regains of the silk/rayon webs and nonwoven fabrics with different silk contents.

Moisture absorption capacity is an essential property of biomedical and cosmetic materials, as it is required for mask packs and wound dressings. The moisture regain of the silk web and nonwoven fabric was measured, and the results are depicted in Figure 4. Overall, the silk/rayon webs showed slightly higher moisture regain values (less than 1%) than the nonwoven fabric. The crystalline structure of natural polymers, such as silk, restricts their water absorption [43]. The heat treatment crystallizes silk and rayon to a lesser extent, as depicted in Figure 2. Therefore, the slight increase in the moisture regains of the nonwoven fabrics compared with webs may be due to the slight additional crystallization of silk and rayon by the heat treatment.

Furthermore, as the rayon content increased, the moisture regains in the silk/rayon web and nonwoven fabric increased. As stated previously, this is because rayon filaments are more hydrophilic than silk filaments. Thus, more hydrophilic rayon absorbs more water and becomes more flexible when the water evaporates (Figure 1).

**Figure 5:**
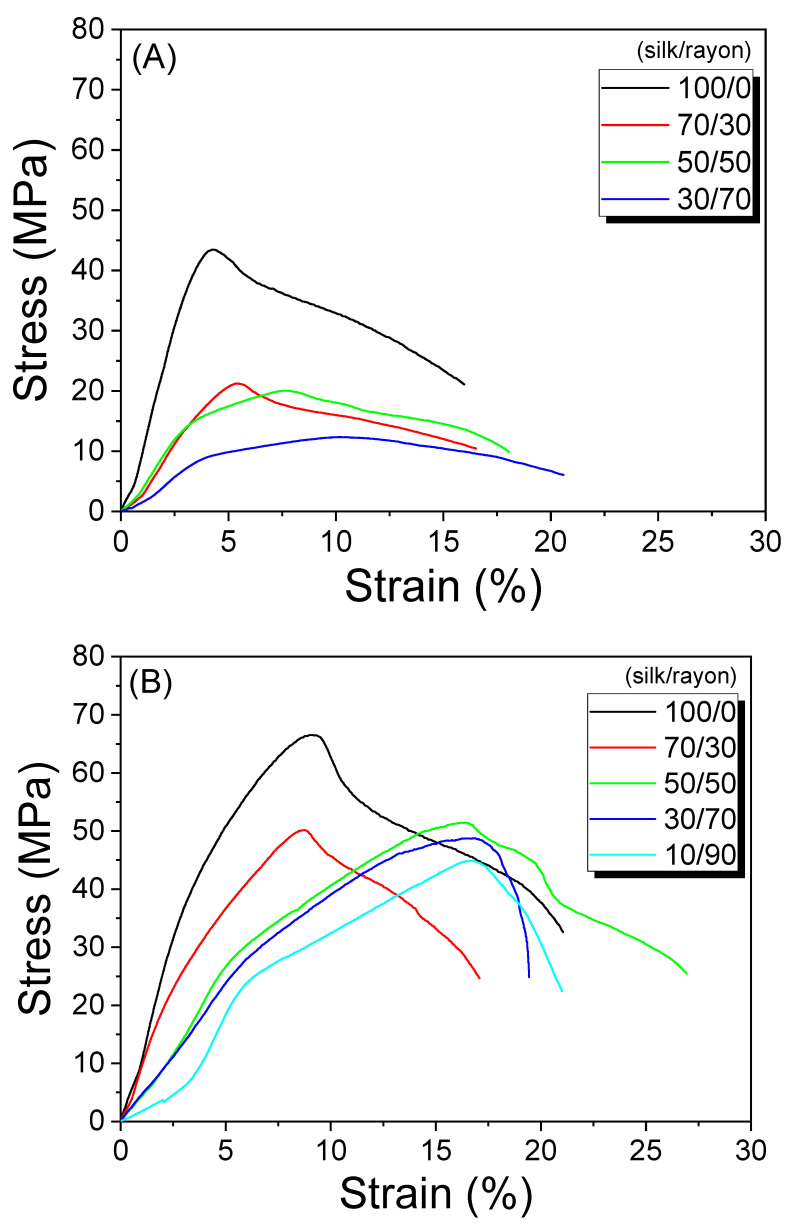

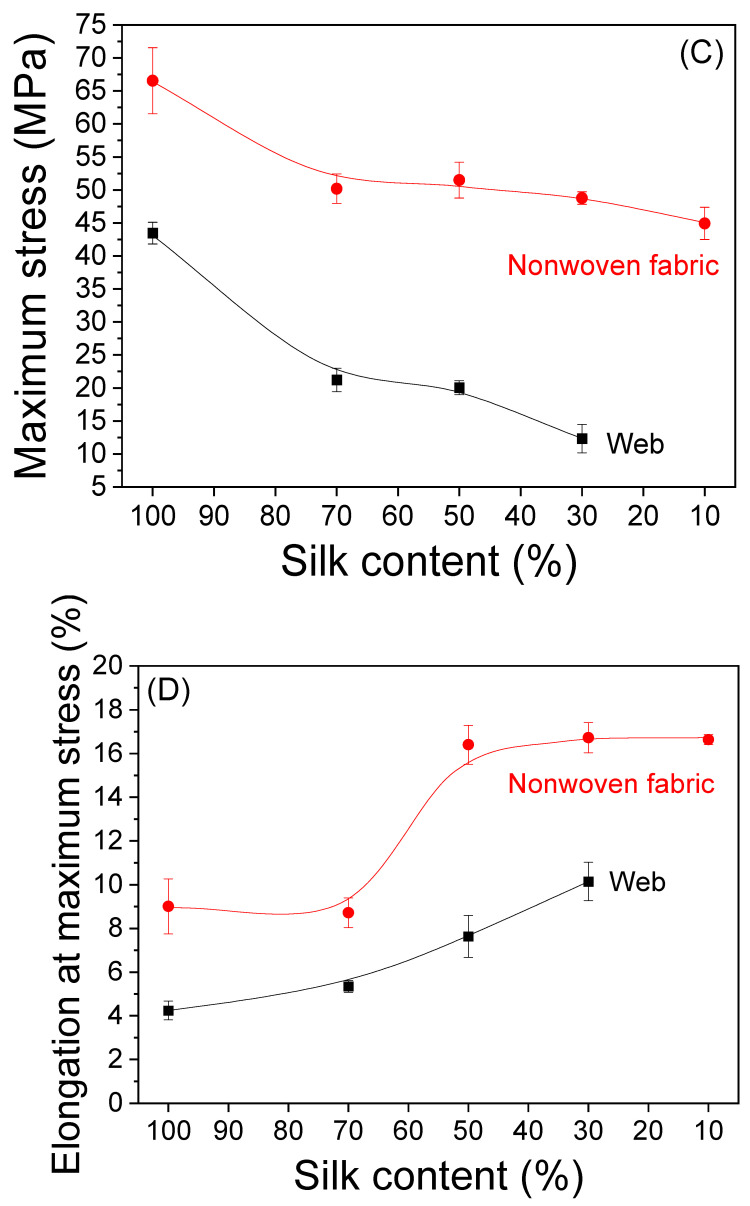
Representative stress-strain curve of (**A**) silk/rayon webs, (**B**) nonwoven fabrics with different silk/rayon compositions, (**C**) maximum stress, and (**D**) elongation at maximum stress of webs and nonwoven fabrics with different silk contents.

Figure 5 shows the effect of the silk/rayon composition on the mechanical properties of the web and nonwoven fabric. Silk/rayon nonwoven fabrics exhibited greater maximum stress and elongation at the maximum stress than silk/rayon webs, regardless of the silk/rayon composition. This is because wet and hot-press treatments increase the binding strength of sericin. The wet treatment swells the sericin, and the hot-press treatment increases its binding effect [24,25,26]. This causes the stress and elongation of the nonwoven fabric to increase in comparison to the web.

In addition, as the silk content increased, the maximum stress of the web and nonwoven fabric increased, and their elongation at the maximum stress decreased. This outcome may be attributable to the nature of each component (i.e., silk and rayon). The silk filament is more rigid (less ductile) than the rayon filament. Therefore, as the amount of the stiffer material (silk) increases, the maximum stress of silk/rayon webs and nonwoven fabrics increases, and their elongation decreases (Figure 5C,D). The binding effect of sericin may also contribute to this behavior. As the amount of silk increases, the amount of binder (sericin) increases. This causes the binding force between silk and rayon filaments to increase, resulting in an increase in the maximum stress value based on the silk content. Given the similar trend between the web (weak binding effect of sericin) and nonwoven fabric (strong binding effect of sericin), the binding effect of sericin is considered to have a minor impact on this trend.

**Figure 6:**
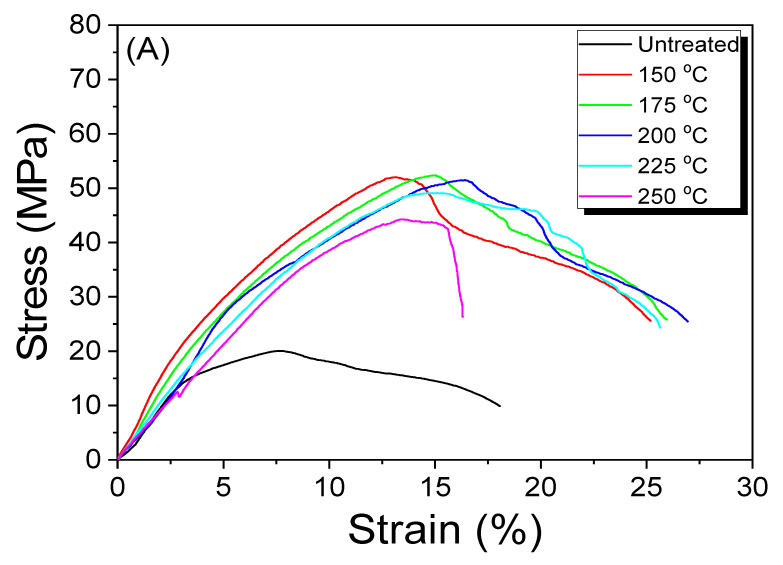

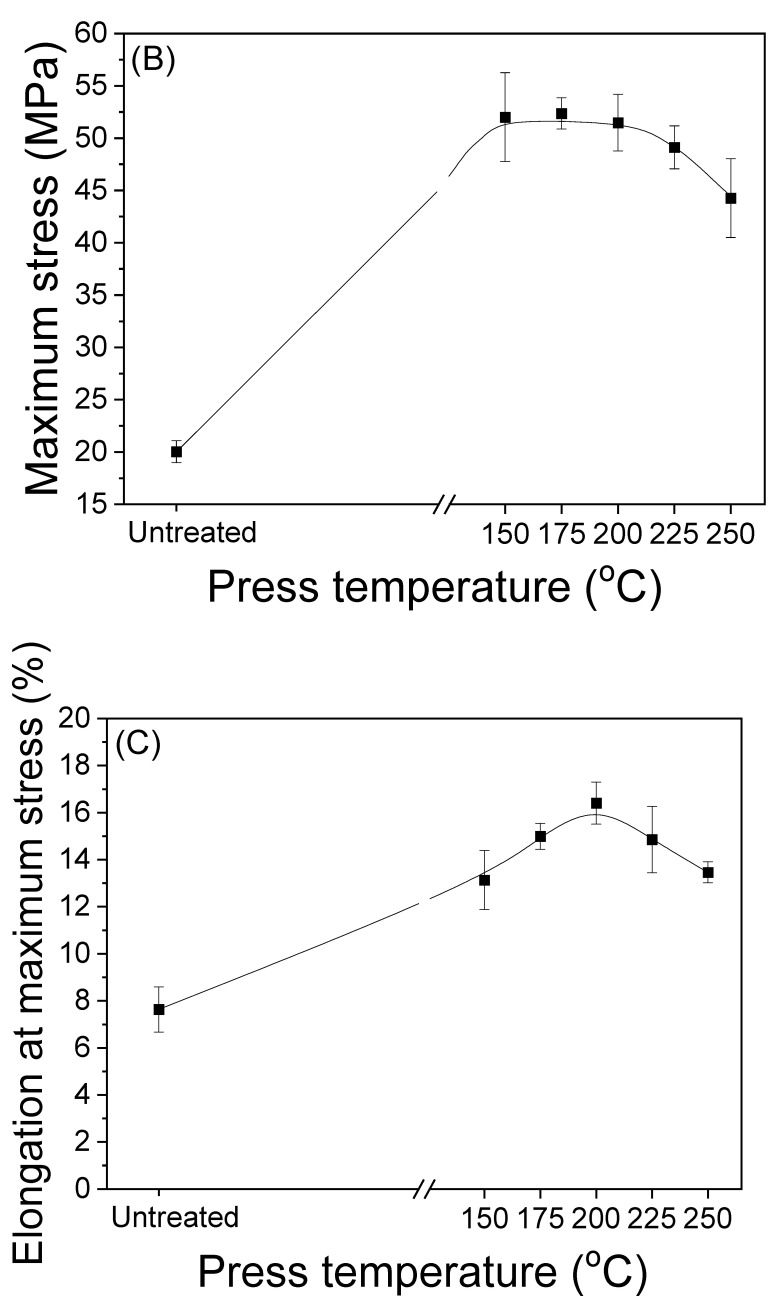
(**A**) Representative stress–strain curve, (**B**) maximum stress, and (**C**) elongation at maximum stress of silk/rayon nonwoven fabrics (50/50) with different press temperatures.

According to reports, the press temperature significantly affects the mechanical properties of silk nonwoven fabric [24,25,31]. Thus, the silk/rayon (50/50) nonwoven fabrics were prepared at different press temperatures to examine the effect of the press temperature on their morphology and mechanical properties. As the press temperature was increased to 150 °C, the maximum stress and elongation at the maximum stress increased significantly and decreased above 200 °C. The mechanical properties of the silk nonwoven fabric improved until 200 °C was reached and then deteriorated. The results presented in Figure 6B,C are consistent with those of previous studies. Lee et al. reported that the binding effect of sericin increases until 200 °C is reached [31]. However, the binding effect of sericin is reduced above 200 °C because thermal degradation of sericin begins at above 200 °C, resulting in a deterioration of mechanical properties [44,45]. As the thermal decomposition of silk fibroin and viscose rayon occurs at approximately 280 °C [33] and 270 °C [46], respectively, the thermal properties of sericin dominate the mechanical properties of silk/rayon nonwoven until a press temperature of 250 °C is reached.

**Figure 7:**
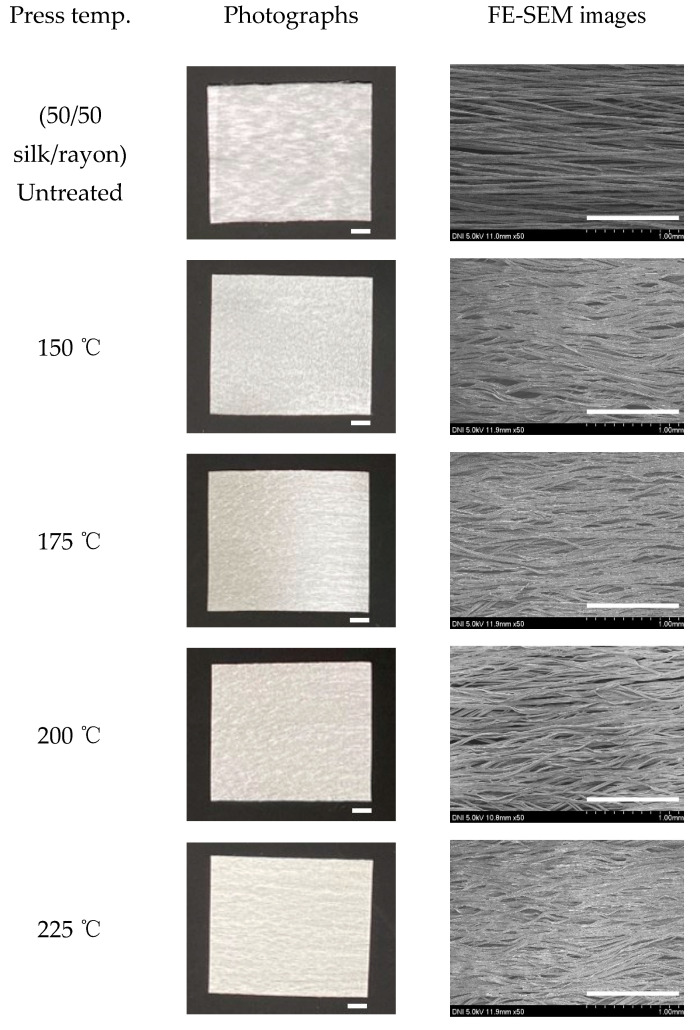

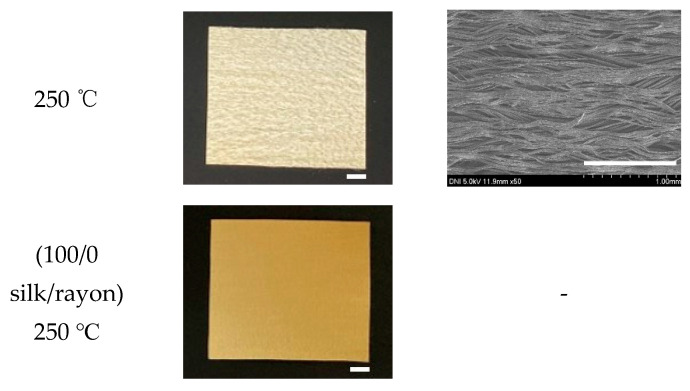
Photographs and FE-SEM images of silk/rayon nonwoven fabrics (50/50) with different press temperatures. The white magnification bars in the photographs and SEM images represent 1.0 cm and 1.0 mm, respectively.

**Figure 8:**
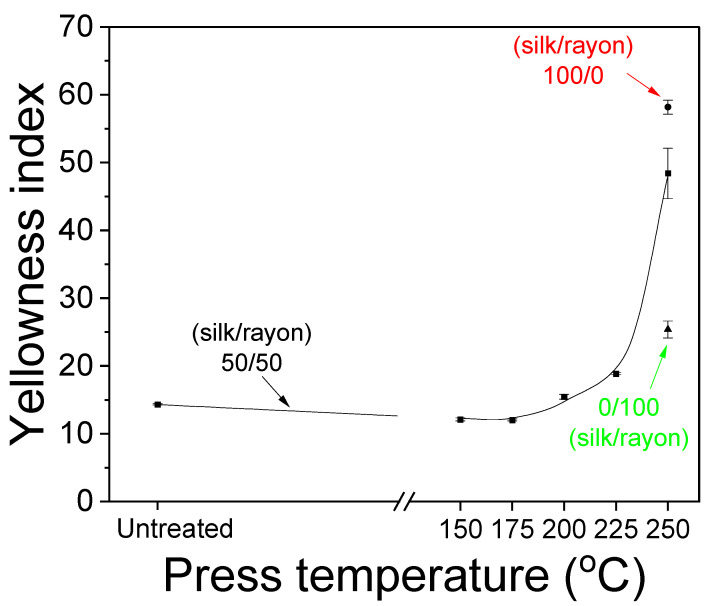
Effect of the press temperature on the yellowness index of silk/rayon (50/50) nonwoven fabrics.

**Figure 9:**
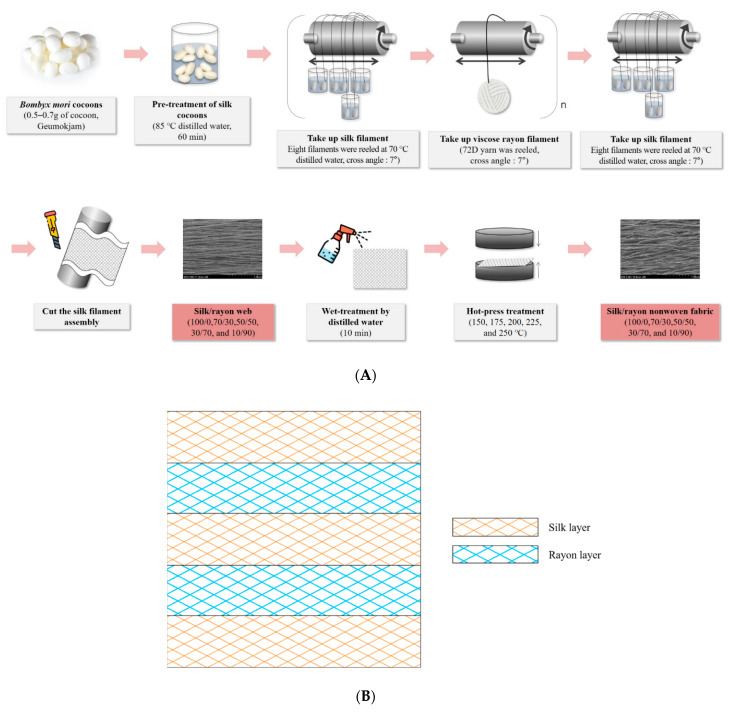
Schematic illustrating (**A**) the preparation of the silk/rayon web and nonwoven fabric and (**B**) the arrangement of five layers in the silk/rayon webs and nonwoven fabrics.

Figure 7 displays photographs and SEM images of the silk/rayon (50/50) nonwoven fabric pressed at various temperatures. The color of the nonwoven fabric did not change as the press temperature was increased to 225 °C. However, at 250 °C, the fabric appeared yellow, owing to the yellowing of silk. This result was consistent with those of previous studies [24,25,31]. Because severe yellowing indicates the thermal damage of silk and cellulose material and the loss of whiteness of material, severe yellowing should be avoided. Thus, the yellowness index was calculated to quantify the degree of yellowing, and the result is presented in Figure 8. The yellowness index of the nonwoven fabric did not change as the press temperature was increased to 175 °C. Subsequently, it increased slightly with an increasing press temperature and increased significantly to 48.4 at 250 °C. A yellowness index of 48.4 at 250 °C implies the severe thermal damage of silk/rayon nonwoven fabric resulting in the loss of mechanical properties, as shown in Figure 6. This means a press temperature of 250 °C should be avoided to fabricate the silk/rayon nonwoven fabric. The yellowness index of silk and rayon was 58.2 and 25.4, respectively, at 250 °C. This indicates that yellowing occurs in both silk and rayon, albeit to varying degrees depending on the material. The yellowness index of the silk/rayon (50/50) nonwoven fabric was closer to that of silk than that of rayon. This can be attributed to the presence of silk in both surface layers of the silk/rayon nonwoven fabric, as shown in Figure 9B.

### 2.3. Cell Viability of Silk/Rayon Nonwoven Fabric

**Figure 10:**
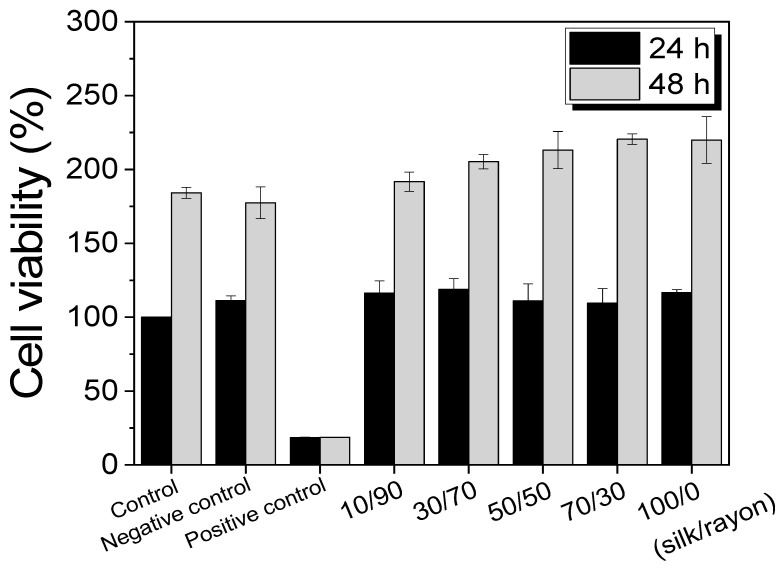
Cell viability of the silk/rayon nonwoven fabrics with different silk/rayon compositions.

As silk/rayon nonwoven fabrics can be used as biomedical and cosmetic products, their cell viability needs to be evaluated. The cell viability of silk/rayon nonwoven fabrics with various silk/rayon compositions was evaluated by using the CCK test, and the results are depicted in Figure 10. In the case of 24 h incubation, regardless of the silk/rayon composition, the cell viability of all silk/rayon nonwoven fabrics was comparable with that of the control (blank) and negative control (HDPE). In addition, after 48 h of incubation, all silk/rayon nonwoven fabrics showed greater cell viability than the control and negative control, despite a slight increase in cell viability with an increasing silk content. This indicates that all silk/rayon nonwoven fabrics have good cell viability, regardless of the silk/rayon composition.

**Figure 11:**
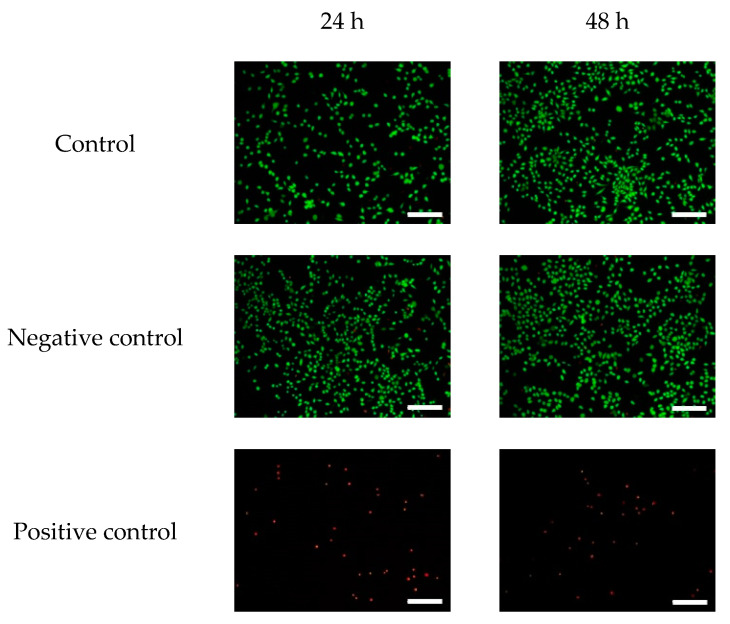

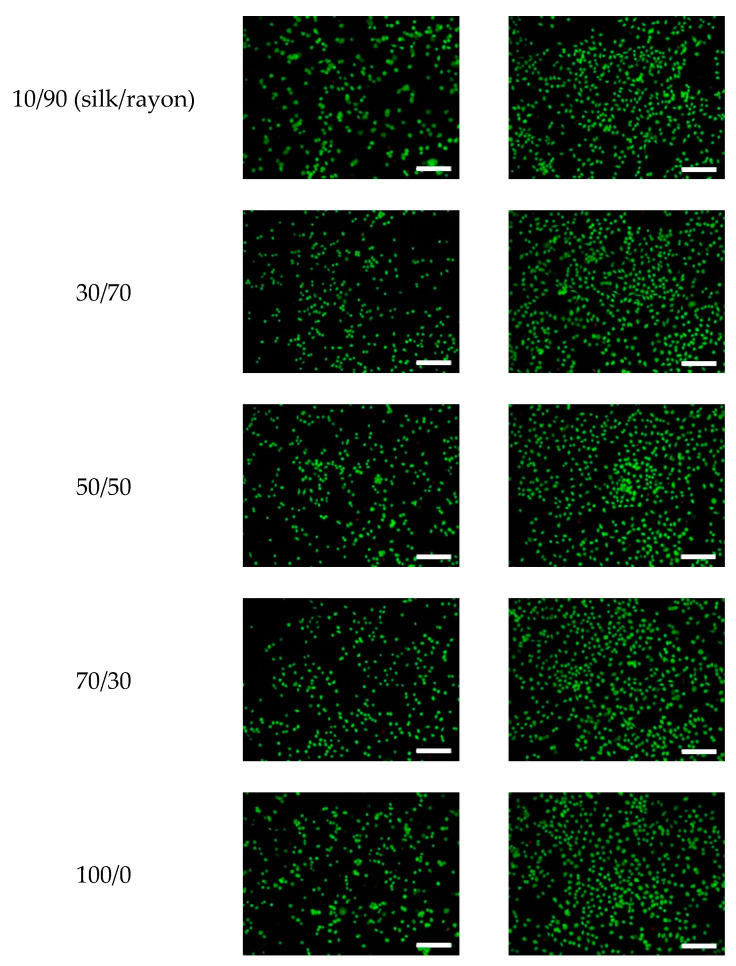
Fluorescence images of the cell viability assay of the silk/rayon nonwoven fabrics with different silk/rayon compositions. The white magnification bars in images represent 200 μm.

The cell image of the silk/rayon nonwoven fabric (Figure 11) confirms the CCK test results. As the incubation period increased to 48 h, more viable cells were observed. Regardless of the silk/rayon composition, all silk/rayon nonwoven fabrics displayed a number of live cells comparable with those of the control and negative control, indicating that the silk/rayon nonwoven fabrics are cytocompatible. These results are easily explicable because silk and rayon exhibit high cytocompatibility [31,47,48].

## 3. Experimental

### 3.1. Materials

Kumokjam *Bombyx mori* silkworm cocoons with dead pupa were provided by Gyeongsangbuk-do Silkworm & Insect Management Center (Sangju, Korea). Among the cocoons, those weighing 0.5–0.7 g were selected and used of comparable lengths. Viscose rayon filaments (72 deniers) were provided by Hansin Co., Ltd. (Yangsan, Korea). The silkworm cocoons and viscose rays were used in the fabrication of the silk/rayon web and nonwoven fabric. L929 mouse fibroblast cells (CCL-1) were provided by ATCC (USA).

### 3.2. Preparation of Silk/Rayon Web and Nonwoven Fabric

The preparation process for the silk/rayon web and the nonwoven fabric is displayed in Figure 9A. The silkworm cocoons were soaked in a distilled water bath at 85 °C for 60 min as a pretreatment to swell the silk sericin within the cocoon. The silkworm cocoons were then transferred to reeling baths at 70 °C prior to the manufacturing of webs by using a silk web manufacturing machine (SNWFM-2, Donga Machinery, Korea). The web manufacturing machine consisted of (1) six electric winders with a controller of reeling speed and a controller of transverse movement and (2) eight reeling baths per winder. The reeling speed (97.5 m/min) and transverse speed (5.6 m/min) were adjusted to produce a silk/rayon web in which the cross-over angle of two silk (or rayon) filaments was 7°. A silk/rayon composite web was manufactured by stacking silk and viscose rayon filaments layer by layer. As shown in Figure 9B, the web consists of a five-layer structure: silk–rayon–silk–rayon–silk. First, eight silk filaments were reeled from the cocoon and wound on the electric winder to form the first layer. The second layer was then formed by winding 72-denier viscose rayon filaments on the winder. This procedure was repeated once more. Finally, silk filaments were wound into a five-layer silk/rayon web structure. For reference, silk filaments were located at the top and bottom layers in the silk/rayon web because locating the viscose rayon at the outer layers of the silk/rayon web was impossible owing to the lack of a binder in rayon. To prepare webs with different silk/rayon (weight) compositions (100/0, 90/10, 70/30, 50/50, 30/70, and 10/90), the amount (weight) of silk and rayon filaments was controlled by varying the reeling time of silk and rayon.

After silk and rayon filaments were wound, silk/rayon webs were obtained by cutting the filament assembly on the roller (winder). The silk/rayon webs were then sprayed with distilled water for 10 min and pressed twice by using a hot presser (HK 2008-1-5, Hankuk Industry Co., Gwangju, Korea) at 150, 175, 200, 225, and 250 °C for 10 s to produce the silk/rayon nonwoven fabric. Smooth polyester nonwoven fabrics were placed on top and bottom of the silk/rayon web to prevent the web from adhering to the hot press plates [23,25,26,31].

### 3.3. Measurement and Characterization

The external features of the silk/rayon web and nonwoven fabric were photographed using a camera (iPhone 11 Pro, Apple Inc., Cupertino, CA, USA). The morphology of the silk/rayon web and nonwoven fabric coated with Pt-Pd was evaluated using field-emission scanning electron microscopy (FE-SEM, S-4800, Hitachi, Tokyo, Japan).

The crystalline structures of the silk/rayon web and nonwoven fabric were determined using the wide-angle X-ray scattering (WAXS) method. X-ray diffraction (XRD) with *2θ* scanning was performed by employing a Micro X-Ray scattering system (D8 Discover, Bruker, Karlsruhe, Germany) using Cu *Kα* radiation. The irradiation conditions were 50 kV and 1000 μA, and the measurement time was 300 s [49].

To determine the porosity of the silk/rayon web and nonwoven fabric, the web and fabric were immersed in ethanol of volume V_1_ for 5 min. After the silk/rayon sample was completely soaked and the ethanol permeated the sample, the total volume (V_2_) of ethanol and the sample was measured. The silk/rayon sample was extracted from the ethanol, and the residual ethanol volume (V_3_) was recorded. The porosity of the sample was then calculated by applying Equation (1) [25,50]. This method was used to determine porosity three times for each sample, and the average porosity of the silk/rayon samples was reported.

(1) $$\mathrm{Porosity}\;(\%)=\frac{V_{1}-V_{3}}{V_{2}-V_{3}}\;\times \;100$$

To determine the moisture regain of the silk/rayon web and nonwoven fabric, they were kept under standard conditions (20 °C and 65% relative humidity (RH)) for 24 h, and the moisture regain of the silk/rayon sample was calculated using Equation (2). The dry weight of the silk/rayon sample was determined with a moisture-balance instrument (XM60, Precisa Gravimetrics, Dietikon, Switzerland).

(2) $$\mathrm{Moisture}\;\mathrm{regain}\;(\%)=\frac{\mathrm{Initial}\;\mathrm{weight}\;-\;\mathrm{Dry}\;\mathrm{weight}}{\mathrm{Dry}\;\mathrm{weight}}\;\times \;100$$

To evaluate the mechanical properties of the silk/rayon web and fabric, the maximum stress and elongation at the maximum stress were measured using a universal testing machine (OTT-003, Oriental TM, Ansan, Korea). In the case of these silk samples [23,25,31], the test was conducted by using a 200 kgf load cell at an extension rate of 10 mm/min and a gauge length of 50 mm. The samples were cut into 50 mm × 30 mm pieces and preconditioned at 20 °C and 65% RH. Seven samples were tested for each condition, and the average and standard deviation of the measurement results were calculated from the five results after the maximum and minimum values were removed.

The color of the silk/rayon nonwoven fabric was quantitatively evaluated by using the CIE 1931 color space. CIE tristimulus (XYZ) values were determined based on CIE standard illuminant D65 and the specular component excluded (SCE) mode of the colorimeter (Konica Minolta, CR-300 Chroma meter, Osaka, Japan). The yellowness index was calculated by applying Equation (3) [51].

(3) $$\mathrm{Yellowness}\;\mathrm{index}=\frac{1.28X-1.06Z}{Y}$$

L929 cells were grown in a RPMI1640 medium (Gibco) supplemented with 10% (*v/v*) fetal bovine serum and 1% (*v/v*) antibiotic-antimytotic solution. The L929 cells were incubated at 37 °C in a humidified 5% CO_2_ atmosphere. When 80% confluence was observed, subculture was performed twice per week.

The in vitro cytotoxicity test of the sample was performed by employing an extract method in accordance with ISO 10993-5. Before extraction, each sample was sterilized with E.O. gas. The extraction was performed by immersing the samples (6 × 3 cm^2^) in the 6 mL RPMI1640 culture medium with gentle shaking at 37 °C for 24 h. The ratio of the sample surface area to the extraction vehicle volume was 3 cm^2^/mL. Latex and HDPE, respectively, served as the positive and negative controls.

The cytotoxicity of the samples on the L929 cells was determined by applying the CCK-8 (Cell Counting Kit 8, Dojindo, Japan) assay in vitro. The L929 cells were seeded into 96-well plates at 1 × 10^5^ cells/mL ratio and incubated at 37 °C for 24 h in a 5% CO_2_ atmosphere. The culture medium was then replaced with 100 µL/well of sample extracts. After 24 h and 48 h of incubation, the extracts were discarded for CCK assay, and 100 µL of a 10% (*v/v*) CCK-8 solution was added to each well. After 1 h of incubation, the absorbance was measured at 450 nm. Subsequently, the cell viability of the silk/rayon nonwoven fabric was calculated using Equation (4).

(4) $$\mathrm{Cell}\;\mathrm{viability}\;(\%)=\frac{\mathrm{Absorbance}\;\mathrm{of}\;\mathrm{the}\;\mathrm{test}\;\mathrm{sample}}{\mathrm{Absorbance}\;\mathrm{of}\;\mathrm{the}\;\mathrm{control}}\;\times \;100$$

The cytotoxicity was evaluated by performing fluorescence staining using a live/dead viability/cytotoxicity kit (L3224, Invitrogen, Waltham, MA, USA), as per the manufacturer’s protocol. The L929 cells were seeded into 24-well plates at 1 × 10^5^/mL ratio and incubated for 24 h in a 5% CO_2_ atmosphere at 37 °C. Then, the culture media was replaced by 300 µL/well of sample extracts. After 24 h and 48 h of incubation, the extracts were discarded, and 300 µL of a staining solution was added to each well. After 45 min of incubation, the staining solutions were removed, and the cells were observed using a fluorescence inverted microscope (IX83, Olympus, Tokyo, Japan).

## 4. Conclusions

In this study, silk/rayon composite nonwoven fabrics were fabricated by winding, wetting, and hot-pressing, and the effect of the silk content on the structure and properties of the fabrics was examined. Combining the two components had no effect on the crystalline structure of silk and rayon. As the rayon content of nonwoven fabrics increased, their porosity and moisture retention increased. Hot-press treatment substantially increased the maximum stress and elongation of silk/rayon webs. As the silk content decreased, the maximum stress decreased, and the elongation increased. At approximately 200 °C, the maximum stress and elongation values of the fabric were the highest. At more than 225 °C, significant yellowing of the fabric occurred. Regardless of the silk/rayon composition, the silk/rayon nonwoven fabric exhibited good cell viability.

Utilizing rayon filaments can considerably increase the productivity and production cost of silk nonwoven fabrics. Additionally, the morphological structure of the nonwoven fabric (i.e., porosity), hydrophilicity, and mechanical properties can be diversely controlled by manipulating the silk/rayon composition while maintaining good cytocompatibility of silk. Therefore, these new nature-based nonwoven fabrics are promising materials for cosmetic and biomedical applications. The fabrication of additional silk-based nonwoven fabrics is expected to increase the future use of silk-based materials in bio-related fields.

## Data Availability

The data presented in this study are available on request from the corresponding author.
